# Elementary Pulmonary Rehabilitation Protocol to Ameliorate Functionality Level in Case of Pneumothorax Following Emphysema: A Case Report

**DOI:** 10.7759/cureus.31421

**Published:** 2022-11-12

**Authors:** Ritika S Bhagwani, Vaishnavi Yadav, Shubhada R Dhait, Samruddhi M Karanjkar, Roshni R Nandanwar

**Affiliations:** 1 Department of Cardiorespiratory Physiotherapy, Ravi Nair Physiotherapy College, Datta Meghe Institute of Medical Sciences, Wardha, IND; 2 Department of Physiotherapy, Ravi Nair Physiotherapy College, Datta Meghe Institute of Medical Sciences, Wardha, IND

**Keywords:** smoking, rehabilitation, dyspnea, emphysema, pneumothorax, chronic obstructive pulmonary disease

## Abstract

Chronic obstructive lung disease (COPD) has been reported as the third leading cause of death globally. The main risk factor for a significant portion of emphysema patients is tobacco use. Additionally, occupational exposure to wood dust enhances the risk of acquiring respiratory disorders, since the respirable wood dust settles into the bronchioles and alveoli and causes lung irritation which presents symptoms like mucus hypersecretion and breathlessness. A secondary complication, emphysema-induced pneumothorax, in the elderly requires the medical intervention of intercostal drainage (ICD) to allow the leak of air out of the thoracic cavity. In this article, we present a case of a 65-year-old male who visited the respiratory department with complaints of breathlessness, fever, and cough with expectoration for four days. He reports a history of tobacco smoking for 30 years with prior hospitalization seven years ago with similar complaints. The patient was initially diagnosed with pulmonary emphysema, which later progressed to spontaneous pneumothorax. He underwent medical management with ICD, which was successful. Following this, an integrated rehabilitation program using various breathing strategies was established in order to get the patient back to his regular daily activities with minimal signs of exhaustion or dyspnea. This protocol proved to be successful in enhancing the patient's respiratory condition.

## Introduction

Emphysema is defined as an abnormal permanent enlargement of the air space distal to the terminal bronchioles, accompanied by degradation of the alveolar walls [1]. Based on the pathologic pattern, emphysema is typically categorized into three categories: centrilobular, panlobular, and paraseptal. The secondary lobule's central airways enlarge with normal distal alveolar ducts and sacs in centrilobular emphysema (CLE), a smoking-related condition that frequently affects the higher lobes [2]. In 2019, 3.23 million people perished since chronic obstructive pulmonary disease (COPD) emerged as the third leading cause of death worldwide. About 90% of fatalities occur due to COPD under the age of 70 years especially in low and middle-income countries [3]. Presenting features of emphysema are cough associated with expectoration, dyspnea, reduced chest expansion, declining lung volumes, etc. [4].

Pneumothorax, which can occur spontaneously or as a result of iatrogenic injury or trauma to the lung or chest wall, is the collapse of the lung when air builds between the parietal and visceral pleura inside the chest. The air is outside the lung but inside the thoracic cavity. Consequently, the lung is under pressure, which increases the risk of its collapse and causes the mediastinum to migrate [5]. The migration of air from the lung into the pleural cavity without trauma is known as a spontaneous pneumothorax (SP). Secondary spontaneous pneumothorax (SSP), as opposed to primary spontaneous pneumothorax (PSP), is the term used to describe an SP in a patient with an underlying chronic lung pathology, such as COPD [6]. Spontaneous pneumothorax shows a bimodal age distribution, with the secondary peak including patients aged ≥50 years The most prevalent underlying condition linked to spontaneous pneumothorax in the aged population is pulmonary emphysema [7].

The clinical features of a pneumothorax are sudden onset of dyspnea and pleuritic chest pain, reduced chest excursion on the affected side, an expanded hemithorax on the affected side, diminished breath sounds, no tactile or vocal fremitus, hyper-resonant percussion, etc. [8]. We provide a case of a patient who had an emphysema-induced pneumothorax and underwent medical care, intercostal drainage (ICD), and collateral physiotherapeutic rehabilitation. Intercostal drainage tube insertion and antibiotic chemotherapy continue to be the main treatments for pneumothorax. In order to collect the fluid, blood, and air and for the underlying lung to expand, intercostal tube drainage is employed. It is inserted into the pleural space through the chest wall and is made of flexible plastic. As a result of the accumulation of air, greater respiratory demands appear in the form of dyspnea, a decrease in chest expansion, and faster breathing. Breathing exercises prescribed by a physical therapist are advised because they help with fluid drainage, maintain chest expansion, and reduce dyspnea. Physiotherapists use a range of strategies to increase ventilation for patients with respiratory diseases. Reduced bronchospasm, clearing of lung secretions, regaining full lung expansion, and optimal functional recovery were the objectives of the treatment [9].

## Case presentation

Patient Information

A 65-year-old male, a carpenter by occupation, visited the respiratory department of tertiary care rural hospital with complaints of breathlessness, fever, and cough associated with expectoration that was mucoid in quality for four days. The patient was prior hospitalized seven years ago in a private hospital in Nagpur, India, due to an acute episode of breathlessness suddenly during work and has been experiencing dyspnea since then. X-ray reports showed hyperinflated lungs bilaterally suggestive of pulmonary emphysema. Previous reports suggest dyspnea of grade I-II on Modified Medical Research Council (MMRC) scale. He has a positive history of dust allergy with seasonal variation, exaggerated during winters. The patient claims consuming tobacco products for 20 years and smoking cigarettes for 30 years. The patient consumed 3-4 cigarettes per day. The patient was admitted to our hospital on August 14, 2022, due to acute exacerbation of dyspnea that was evaluated as grade IV on MMRC. Chest X-ray (posterior-anterior view) investigations showed hyperinflated lungs bilaterally along with hyperlucent lung fields. The patient was diagnosed with ipsilateral left-sided pneumothorax secondary to bilateral centrilobular emphysema. ICD was inserted in the left infra axillary in the fourth intercostal space, the air was drained and underwater seal was placed. After the removal of intercostal drainage, post-operative day four, the patient was further referred to the cardiorespiratory physiotherapy department for the removal of excessive secretions, to reduce dyspnea and improve exercise tolerance.

Clinical findings

**Figure 1:**
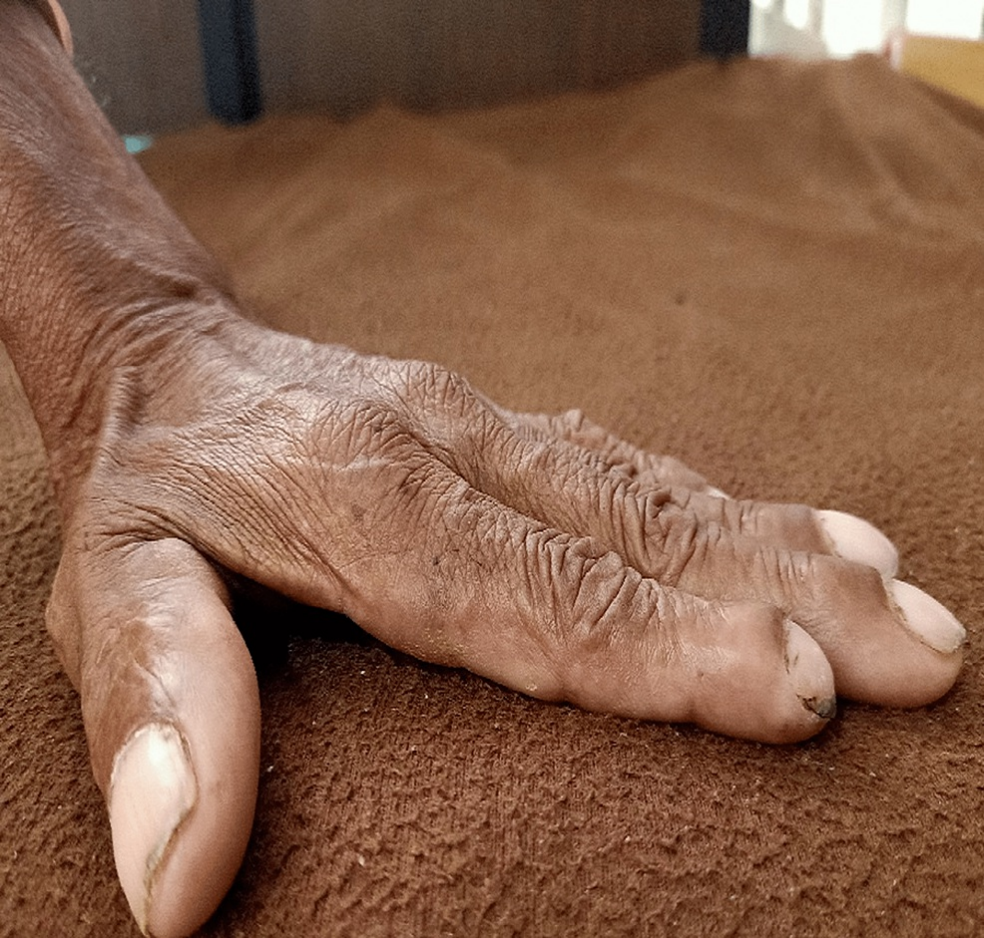
Clubbing (Grade II)

**Figure 2:**
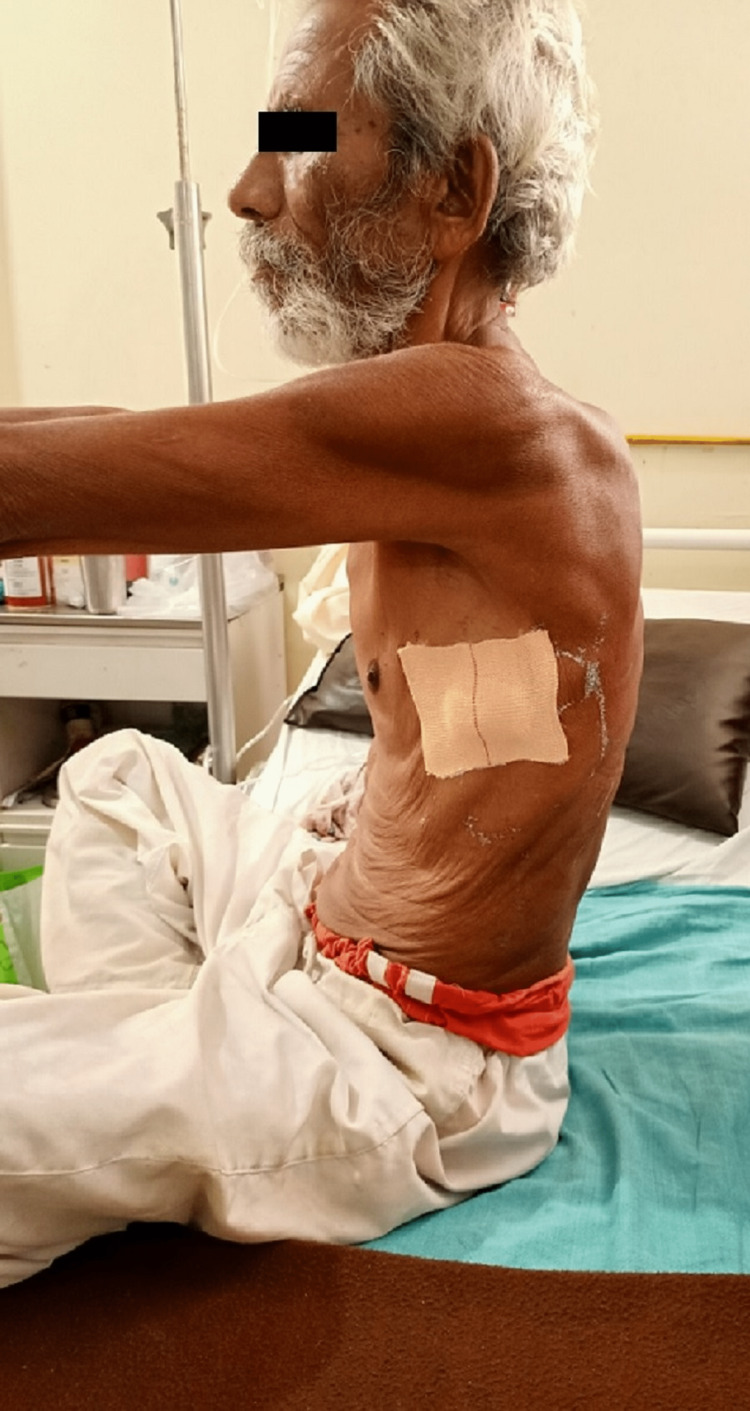
Barrel-shaped chest (anterior and lateral View)

Informed consent was taken from the patient, which was done prior to the physical examination after the removal of ICD. On inspection, the patient was found in a sitting position and was cooperative, with orientation to time, place, and person. Pallor was present, and clubbing grade II was observed with a positive Schamroth window test as shown in Figure 1. From the lateral view, barrel-shaped chest was inspected as shown in Figure 2. The use of accessory muscles was present. On palpation, the trachea was deviated to the right side, chest excursion was found to be reduced bilaterally, and tactile vocal fremitus was diminished in the upper and middle zones. Chest expansion findings were 1cm, 1.5cm, and 2cm at axillary, nipple, and xiphisternal levels, respectively. On percussion, hyper-resonant note was present on the left side, mammary region. On auscultation, breath sounds were diminished in the upper and lower zones on the left side of the thorax.

Diagnostic assessment

**Figure 3:**
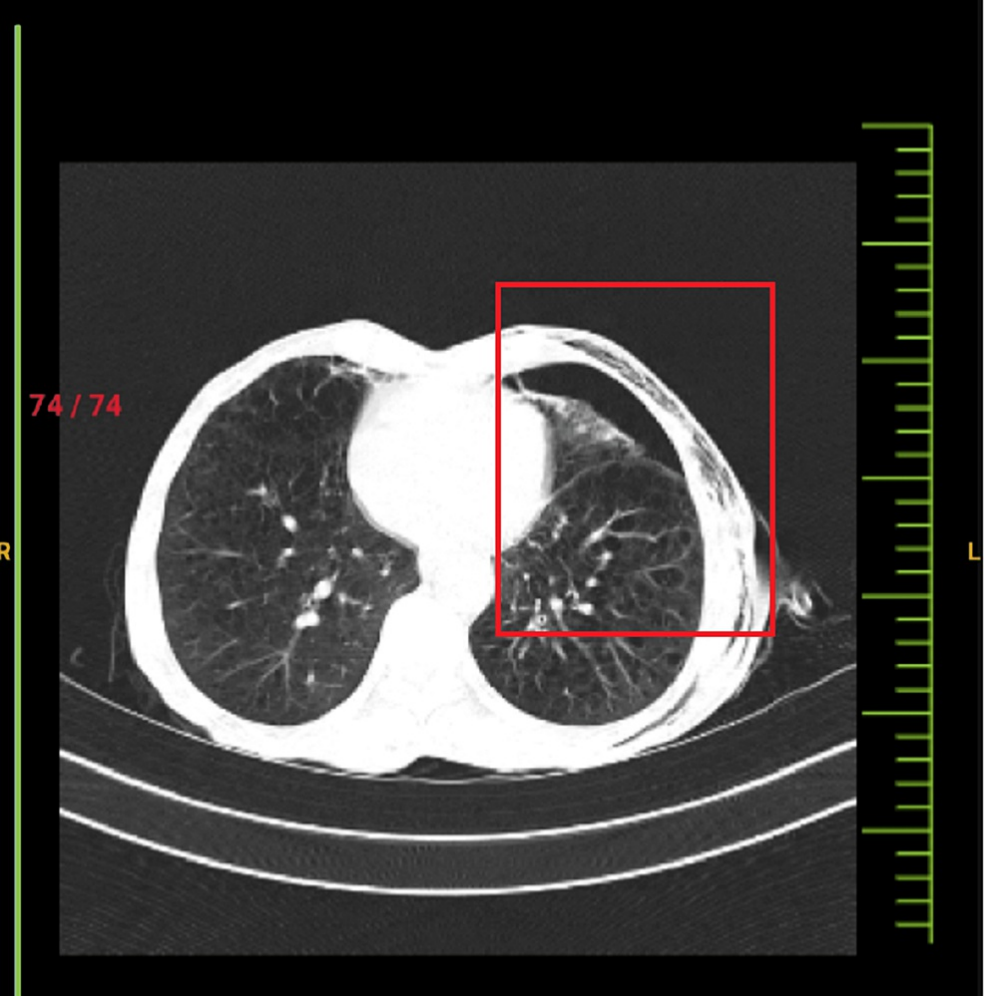
CT Thorax presenting pneumothorax with underlying lung atelectasis

**Figure 4:**
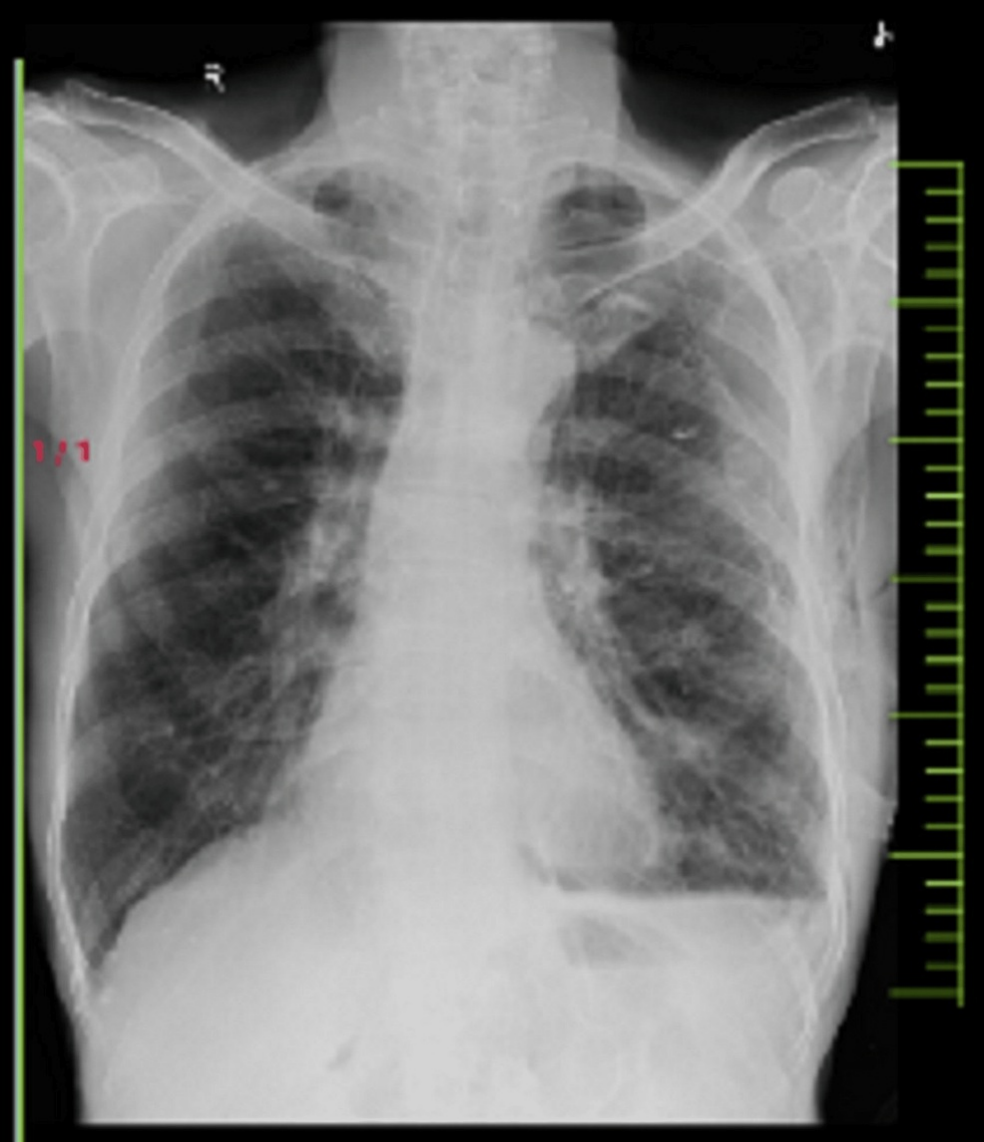
Posterior-anterior view of chest X-ray showing tracheal deviation and flattening of left hemidiaphragm

The patient’s high-resolution CT (HRCT) thorax scan findings revealed ICD with the tip in the left pleural space and a few small areas of fibrotic changes in bilateral lung fields with subsegmental atelectasis. Bilateral lungs appear hyperinflated with centrilobular emphysematous changes, extending superiorly to the ipsilateral axilla, shoulder, and suprascapular region as shown in Figure 3. X-ray findings of the chest revealed hyper lucent lung fields bilaterally, with deviation towards the right side and flattening of the left hemidiaphragm as presented in Figure 4.

Physiotherapy management

Table 1 presents the data of the physiotherapy management strategy that was planned and applied as per the patient's limitations. It enlists the problems faced by the patient, goals, and interventions specific to the problems displayed.

**Table 1:** Physiotherapy Management ICD- Intercostal Drainage; PLB- Pursed Lip Breathing; ACBT- Active Cycle of Breathing Technique; OPEP- Oscillatory Positive Pressure Device; HEP- Home Exercise Program

Serial no.	Problems faced by the patient	Goals	Description of interventions
1.	Pain at the ICD insertion site	To reduce pain	1. TENS: Transcutaneous Electrical Nerve Stimulation was used with the following parameters: Mode: Conventional mode Frequency: 100-150 Hz Duration: 10-15 minutes Site of electrode placement at the site of tube insertion. 2. Splinting technique: The patient was instructed to use a towel pad to support the incision site while coughing, sneezing, laughing etc. 3. Breath stacking exercise (after removal of ICD): The patient was encouraged to breathe in slowly, stacking one breath on top of the other, with five seconds hold; three sessions per day with five repetitions were advised (Mohamed et al., 2021).
2.	Exertional dyspnea	To reduce dyspnea while working	Ventilatory strategies with activity: The patient was explained to breathe through the nostrils and exhale through pursed lips as in blowing a candle. The breathing pattern was synchronized with the activity. For example, explaining to the patient to take two steps and simultaneously inhale and exhale once while walking.
3.	Use of accessory muscles	Provide relaxation to muscles	Dyspnea relieving position with PLB: The patient was instructed to do bedside sitting, place a pillow over both thighs and was asked to lean on it with elbows extended over the pillow. While doing that, the patient was explained to inhale through the nostrils and exhale through pursed lips (five repetitions). Other positions such as sitting on a chair and leaning on the table in front were taught.
4.	Reduced Chest Expansion	To improve chest mobility	Chest Mobility exercises with breathing strategies: 3 different exercises were demonstrated to the patient. He was instructed to sit in a chair and perform pectoral stretch with hold and pectoral stretch while performing trunk rotations. The patient was explained to stand and perform side bends avoiding overstretching with pursed lip breathing. The effectiveness of chest wall mobility lies in its ability to increase ventilation on that side of the chest, emphasize the depth of inspiration, and regulate expiration. [9].
5.	Accumulation of secretions	Clearance of secretions	Active Cycle of Breathing Technique (ACBT): The mechanism of ACBT comprises three exercises- breathing control x3, thoracic expansion exercise x3, forced expiratory exercise consisting of coughing and huffing x2, with breathing control thrice after each exercise. The patient is asked to initiate inhaling and exhaling through nostrils three times, then perform thoracic expansion, with an interval of breathing control, followed by coughing and huffing along with breathing control. This procedure helps the movement of secretions from peripheral to central airways and further to the mouth. Oscillatory positive expiratory pressure (OPEP) device: The patient was given a mechanical hand-held OPEP device, and was told to breathe through it. This device had an inhaling valve and a linear track for the patient to follow. Positive pressure oscillations are caused by a one-way valve opening and closing sporadically during exhale. The patient then coughed and huffed a couple of times. Ten to 20 blows into the device were administered four times per day [10].
6.	Reduced cardiovascular endurance	To improve exercise tolerance	The patient was provided with a well-monitored graded exercise program that started with bedside limb mobility exercises, involved walking, and stair climbing for 10 minutes three to four times per day and was gradually increased in accordance with the patient's hemodynamic response and rate of perceived exertion. Home Exercise Program (HEP): The physiotherapy protocol was designed for two weeks with six sessions a week in the hospital inpatient set-up. Once improvements in his endurance were seen, he was discharged with a well-explained home program. The patient was provided with a plan of exercises that included deep breathing exercises, pursed lip breathing exercise, brisk walking, and stair climbing with symptoms in check and chest mobility exercises.

Outcome measures

Table 2 lists the results that were utilized to evaluate the patient's progress on the day of referral, on the day of discharge, and on the day of follow-up. The patient was given a set of training regimens to follow at home in order to restore strength and increase endurance, including instructions for self-monitoring vital signs and spotting warning signs. Along with this routine, he was also instructed to practice breathing retraining and cleanliness every day, as well as to use relaxation and dyspnea-relieving techniques as needed. Additionally, he was told to follow up after two weeks and contacted by phone if he had any questions about the medication or his health. Positive rehabilitation outcomes were reported by the patient with great satisfaction.

**Table 2:** Outcome measures recorded during the course of treatment and follow-up

Outcome	First day of referral	On the day of discharge	At the time of Follow-up
Functional Independence Measure (FIM)	4	6	7
Six-Minute Walk Distance (6 MWD)	230 m with rest pause	260 m with rest pause	290 m
St. George Respiratory Questionnaire (SGRQ)	50	30	20

Timeline of all the events

Table 3 presents the duration of all the events such as the date of admission, date of surgery, date of physiotherapy referral, date of discharge, and date of the last follow-up.

**Table 3:** Timeline of all the events

Events	Dates
Date of Admission	August 10, 2022
Date of Surgery	August 13, 2022
Date of Physiotherapy Referral	August 17, 2022
Date of Discharge	August 30, 2022
Date of the last follow-up	September 28, 20/22

## Discussion

Chronic respiratory diseases impact an individual’s breathing and ventilation capacities negatively. A deadly disease, COPD, leads to functional impairments and secondary complications like pneumothorax, pulmonary hypertension, pneumonia, and chronic atelectasis following emphysema, the most common form of COPD to prevail. This case study describes a case of an elderly male patient who developed pneumothorax secondary to pulmonary emphysema. He underwent ICD insertion following which intensive physiotherapy care was required to improve the oxygenation and ventilation capacities of the lungs. Breathing exercises include diaphragmatic breathing and segmental expansion as well as pursed lip breathing that creates back pressure over the alveoli, hence allowing prolonged perfusion and the development of collateral channels for ventilation. Conclusion derived from a study on two groups of COPD participants using flutter as well as active cycle of breathing technique (ACBT) for two weeks showed drastic results, by increasing mucus clearance and improving the viscoelasticity of lungs. An increase in peak expiratory flow rate (PEFR) and forced expiratory volume/forced vital capacity (FEV1/FVC) was observed simultaneously [11].

Dimitrova’s research developed a pulmonary rehabilitation protocol to assess the severity of symptoms of COPD pre- and post-intervention using indicators such as six-minute walk test and modified medical research council (MMRC) scale. Aerobic exercises such as walking, slow running, cycling, etc., along with breathing exercises, forced exhalation, and coughing manoeuvres caused symptoms to be less pronounced [12]. To clear out the secretions, the patient in this case study was provided with an oscillatory expiratory pressure device, which he was instructed to blow in 10-20 times, four times each day. Chest recovery was faster and reflected a reduction in symptoms of dyspnea as well. By the outcome measures, improvement was seen in the six-minute walk test, indicating improvement in exercise tolerance. As observed in the findings of a study by Sarah et al., in COPD patients, oscillatory expiratory positive pressure (OPEP) medication significantly improved forced vital capacity (FVC), six-minute walk distance (6MWD), and symptoms in sputum-producers. It also became easier to cough up sputum. Additionally, significant improvements in the St. George respiratory questionnaire (SGRQ), forced expiratory volume (FEV1), 6MWD, and ventilation-perfusion deficit (VDP) were seen in half of those who generated sputum [10]. Nutsupa et al. in their study showed that patients underwent light exercise training to become acquainted with breathing regulation while doing activities of daily life after ventilatory feedback and exercise training that demonstrated a positive impact on ventilation [13].

## Conclusions

The goal of this study was to plan a management structure for a patient of pneumothorax secondary to emphysema in aspects of pulmonary rehabilitation. Prior to the initiation of physiotherapy management, the patient had reduced oxygen saturation and poor mucus clearance. However, our approach with an integrated and comprehensive rehabilitation regimen showed positive changes in the severity of symptoms of dyspnea, cough, mucus clearance, pulmonary capacities, weakness, and overall quality of life. It is vital for these parameters to resolve in elderly patients so that they can get back to their pre-disease state and spend the rest of the years of their lives in peace and prosperity.
